# The role of epistemological beliefs in STEM faculty’s decisions to use culturally relevant pedagogy at Hispanic-Serving Institutions

**DOI:** 10.1186/s40594-022-00349-9

**Published:** 2022-04-12

**Authors:** Mollee Shultz, Jayson Nissen, Eleanor Close, Ben Van Dusen

**Affiliations:** 1 Physics Department, Texas State University, 601 University Dr., San Marcos, TX 78666 USA; 2 Nissen Education Research and Design, 2565 NW Lincoln Ave, Corvallis, OR 97330 USA; 3 School of Education, Iowa State University, Lagomarcino Hall, 901 Stange Road, Ames, IA 50011 USA

**Keywords:** Culturally relevant pedagogy, Epistemology, Beliefs, Professional obligations, Hispanic-Serving Institutions

## Abstract

**Background:**

The growing understanding of the oppressive inequities that exist in postsecondary education has led to an increasing need for culturally relevant pedagogy. Researchers have found evidence that beliefs about the nature of knowledge predict pedagogical practices. Culturally relevant pedagogy supports students in ways that leverage students’ own cultures through three tenets: academic success, cultural competence, and sociopolitical consciousness. If STEM practitioners believe that their disciplines are culture-free, they may not enact culturally relevant pedagogy in their courses. We investigated how and in what forms 40 faculty from mathematics, physics, chemistry, and biology departments at Hispanic-Serving Institutions enacted culturally relevant pedagogy. We used the framework of practical rationality to understand how epistemological beliefs about the nature of their discipline combined with their institutional context impacted instructors’ decision to enact practices aligning with the three tenets of culturally relevant pedagogy.

**Results:**

In total, 35 instructors reported using practices that aligned with the academic success tenet, nine instructors with the cultural competence tenet, and one instructor with the sociopolitical consciousness tenet. Instructors expressed and even lauded their disciplines’ separation from culture while simultaneously expressing instructional decisions that aligned with culturally relevant pedagogy. Though never asked directly, six instructors made statements reflecting a “culture-free” belief about knowledge in their discipline such as “To me, mathematics has no color.” Five of those instructors also described altering their teaching in ways that aligned with the academic success tenet. The framework of practical rationality helped explain how the instructors’ individual obligation (to the needs of individual students) and interpersonal obligation (to the social environment of the classroom) played a role in those decisions.

**Conclusions:**

Instructors’ ability to express two contradictory views may indicate that professional development does not have to change an instructor’s epistemological beliefs about their discipline to convince them of the value of enacting culturally relevant pedagogy. We propose departmental changes that could enable instructors to decide to cultivate students’ cultural competence and sociopolitical consciousness. Our findings highlight the need for future research investigating the impacts of culturally relevant pedagogical content knowledge on students’ experiences.

## Introduction

Scholarship has explored how culturally relevant pedagogy (CRP; Ladson-Billings, 1995, 2014) can be leveraged to make science, technology, engineering and mathematics (STEM) classrooms more equitable, inclusive spaces for increasingly diverse student populations (Gay, 2002; Johnson, 2011; Johnson & Elliot, 2020; Kokka, 2019; Morrison et al., 2008; Ukpokodu, 2011). Beyond reducing racial achievement gaps (Byrd-Wright, 2020), studies have found that various forms of CRP improved students’ attitude and interest towards science and mathematics (Edmin & Lee, 2012; Hubert, 2014) and improved students’ mathematical literacy (Gradini & Firmansyah, 2020). However, researchers have documented the difficulty of implementing culturally relevant pedagogies or multicultural education (Cochran-Smith, 2004; Sleeter et al., 2004), particularly in mathematics and sciences (Gay, 2002; Gay & Howard, 2000). For some instructors, culturally relevant pedagogy is applicable to other subjects but not to STEM fields (Brown et al., 2019).

Among undergraduate institutions, teaching in a way that leverages students’ backgrounds and strengths is particularly important at Minority-Serving Institutions (MSIs). The National Academies of Science, Engineering and Medicine (NASEM, 2019) identified MSIs as an underutilized resource for strengthening STEM fields. This study is conducted with instructors from a range of Hispanic-Serving Institutions (HSIs), a subset of MSIs. Postsecondary institutions with an enrollment of at least 25% or more Hispanic undergraduate full-time students can apply for HSI status (U.S. Department of Education, 2021). Unlike some MSIs, such as Historically Black Colleges and Universities, HSIs are not formed with the primary mission of serving their Hispanic demographic. Given this lack of intentionality behind the formation of HSIs, faculty may not be philosophically aligned with the Hispanic-serving nature of the institution. Faculty may have been hired well before the institution earned HSI status. Little is known about what instructors at HSIs believe or practice in terms of addressing the needs of their diverse student populations.

Given the potential for CRP to progress STEM education, we aim to understand how epistemological beliefs play a role in instructional decisions to use such pedagogies in the HSI context. Researchers have found evidence that beliefs about the nature of knowledge predict pedagogical practices (e.g., Martínez-Sierra et al., 2020; Raymond, 1997). We agree with Ladson-Billings (1997) that “[Culture] informs all human thought and activity and cannot be suspended as human beings interact with particular subject matters or domains of learning” (p. 700). While education researchers have expressed that the teaching of STEM subjects is an inherently political activity (Gutiérrez, 2017; Mendick, 2011; Prescod-Weinstein, 2020), many STEM instructors still express beliefs that their fields are objective and free of culture (Miller-Young et al., 2018; Robertson & Elliot, 2020). Little research has investigated how or why STEM instructors teach with CRP at HSIs, and how beliefs about the nature of disciplinary knowledge might play a role in those decisions.

In this study, we use the framework of practical rationality for teaching (Herbst & Chazan, 2003, 2012) to gain an understanding of how STEM instructors at HSIs make instructional decisions. In particular, we aim to explore if and how STEM instructors enact CRP and the reasons instructors articulate for choosing to do so, given their epistemological beliefs and the communities that they serve. Using the theoretical grounding of practical rationality allows us to productively examine what barriers or incentives exist towards making instructional changes.

## Literature review

In this section, we review the literature on beliefs generally, and then focus on epistemological beliefs about science and mathematics disciplines. We then review literature on culturally relevant pedagogy (CRP) and draw connections between how epistemological beliefs might motivate or hinder use of CRP. These literatures drove our choice of the practical rationality framework that follows.

### Beliefs

Beliefs have taken a central role in education research due to their theorized importance in guiding decision-making (e.g., Bandora, 1986; Bryan, 2012; Dewey, 1933). Researchers have been concerned that teachers hold beliefs that may be incompatible with recommendations for reform (Battista, 1994). Beliefs might be resistant to intervention because researchers have found evidence that beliefs are formed over time through experiences (Raymond, 1997; Nespor, 1987). Abelson (1979) scrutinized the differences between beliefs and knowledge, and contended that, unlike knowledge, beliefs were formed through personal, episodic experience, in addition to cultural folklore and political propaganda. Similarly, Nespor (1987) observed that teacher beliefs were shaped by “crucial experiences” (p. 333) and especially influential teachers that inspired and provided a template for their own pedagogical practices. Buchmann (1987) contended that students expect to become teachers like teachers they have known, and Lortie (1975) observed that teachers usually had already developed most of their beliefs from their own experiences as students. In the context of HSI instruction, instruction based solely on an instructor’s beliefs could alienate students if instructors’ experiences are fundamentally different from their students’. A subset of the beliefs instructors hold that might influence their teaching are those about the nature of the knowledge in their discipline.

### Epistemological beliefs

Researchers have concluded that instructors’ beliefs about the nature of their discipline guide their beliefs about teaching and learning the discipline’s content (Beswick, 2012; Cross, 2009; Ernest, 1989; Johnson, 2011; Martínez-Sierra et al., 2020). In turn, researchers have found that those beliefs about the teaching and learning of science or mathematics have a direct connection with their science or mathematics teaching practices (Bryan, 2012; Cross, 2009; Raymond, 1997; Shultz, 2020). For example, Cross (2009) concluded that teachers draw primarily from their beliefs about the nature of mathematics to inform their beliefs about teaching and student learning from her study of five secondary teachers. She observed that instructors’ view of mathematics as a series of computations or a way of thinking translated into the kinds of activities they designed and the ways they interacted with their students. Martínez-Sierra et al. (2020) found that instructors’ beliefs about the nature or purpose of mathematics were central to their belief systems about teaching, learning and assessment.

A core set of beliefs instructors hold are the beliefs about what constitutes knowledge in their disciplinary field. STEM disciplines have been praised for the objectivity of knowledge that the fields produce. In science and mathematics, some instructors think their subjects are incompatible with teaching about cultural diversity (Gay, 2002). Traweek (1988) described the work of doing particle physics as “an extreme culture of objectivity: a culture of no culture, which longs passionately for a world without loose ends, without temperament, gender, nationalism” (p. 162).

This belief about the absolute objectivity of knowledge may not be equivalent across the sciences and mathematics. The belief may be more rigid for mathematics instructors than for instructors in other STEM fields. In a comparison of science and mathematics epistemologies, Develaki (2020) explained that deductive reasoning and logic are both fundamental to proving knowledge in both. The difference is that in pure mathematics, deductive reasoning and logic are paramount—whereas in the sciences, that is the starting point to then test and confirm with empirical evidence. Perhaps due to this difference, Rabin et al. (2021) found that chemists, biologists, and physicists, in a study with mathematicians, noted that the mathematicians’ thinking was too abstract and not based in context or reality.

On the other end of the spectrum, biology may be seen as more culture-dependent than mathematics and the other physical sciences. Mayr (2004) described how the nature of biology is fundamentally distinct from the physical sciences because of the complexity of living beings. Biological systems of microorganisms and cells have rich properties that do not exist in the inanimate world and systems are always open to influence by other systems (Mayr, 2004). An implication of this complexity is that there are not universal natural laws, because so much is up to chance and randomness and because differences in the world cannot be sorted into typologies as in the physical sciences (Mayr, 2004). The things being studied are more context-dependent.

Researchers have found this culture-free epistemological view of STEM fields in instructors’ reflections. In a study where STEM instructors attempted to engage in education research, one of the main challenges they faced was epistemological discomfort (Miller-Young et al., 2018). The instructors described discomfort at the creation of knowledge from data sources that seemed remarkably more subjective than what they were used to, such as from participant self-reports or one-person case studies. Arsac et al. (1992) called this the instructor’s epistemological responsibility—that instructor is accountable for the mathematical meaning that the students make, including the correction of errors and production of acceptable solutions. These reflections show that instructors who hold a culture-free epistemology might struggle with pedagogies that center the students’ experience.

Harding (1998) pushed back that these practices of producing knowledge through the scientific method, with a goal of maximizing objectivity and rationality, are not the “absence of all culture” (p. 61) but instead could be understood as “distinctive cultural features” (p. 61). Various literature in education has demonstrated how knowledge of mathematics and sciences held by people is deeply rooted in culture (Bang & Medin, 2010; Boaler, 1994; Glasson et al., 2010; McKinley, 2005; Medin & Bang, 2014; Ruef et al., 2020) and called for the development of a situated theory of scientific epistemology (Sandoval, 2014). Even in complete abstraction, mathematics requires the construction and selection of representations by people, with affordances and constraints for communication between people (Hefendehl-Hebeker et al., 2019; Hershkowitz et al., 2001). These researchers took the epistemological position that knowledge generated within the disciplines of science and mathematics is not divorced from culture, but rather is profoundly tied to it. Despite this work on the culture of science, we suspect that disciplinary epistemologies rooted in the idea of a culture of no culture may create a tension for STEM instructors to incorporate CRP in mathematics and science content.

### Culturally relevant pedagogy

In addition to the knowledge generated in STEM disciplines themselves being culturally grounded, the teaching and learning of that knowledge is embedded in cultural interactions that are political and racialized (Gutiérrez, 2017; Martin, 2006). What and how people learn is inherently culturally dependent (NASEM, 2018). As an instructor teaches content, the instructor also, at least implicitly, teaches an epistemology along with the content (Brousseau & Warfield, 1999).

To address the teaching of mathematics as a cultural activity, Ladson-Billings (1995) developed the framework of Culturally Relevant Pedagogy (CRP). She wanted to develop a theory that would capture the excellence of practices held by teachers of African-American students in her study. It describes teaching that helps students achieve three outcomes: (1) academic success, “the intellectual growth that students experience as a result of classroom instruction and learning experiences”; (2) cultural competence, “the ability to help students appreciate and celebrate their cultures of origin while gaining knowledge of and fluency in at least one other culture”; and (3) sociopolitcal consciousness, “the ability to take learning beyond the confines of the classroom using school knowledge and skills to identify, analyze, and solve real-world problems”, also known as the three tenets of CRP (Ladson-Billings, 2014, p. 75). She noted that the intersection of culture and teaching had often been approached with a deficit perspective oriented only towards academic achievement, e.g., success measured by grades and standardized test scores. Instead, to foster cultural competence, teachers ought to stress the value of students’ own cultural integrity to foster students' own cultural identities. To foster critical consciousness with CRP, teachers ought to develop the awareness of students to think critically about the systems that produce inequities.

Most often, sociopolitical consciousness is the component that gets the least attention from instructors (Ladson-Billings, 2014). Getting instructors to recognize the importance of sociopolitical consciousness is the most challenging (Ladson-Billings, 2006), as compared to getting instructors to leverage students’ culture, because instructors often have not developed their own sociopolitical consciousness. Though teachers might hold strong opinions about sociopolitical issues, few know much in depth about the issues that directly affect their students’ lives (e.g., employment, health care, housing; Ladson-Billings, 2006).

The epistemological stance of CRP as outlined by Ladson-Billings (1995) is wholly distinct from Traweek’s (1988) observation of a ‘culture of no culture’ within particle physics. Instructors teaching with CRP-treated knowledge as socially constructed and dynamic (Ladson-Billings, 1995). For example, the teachers positioned students as knowledge-creators by asking the students to identify an area that they believed they held expertise in and present it to the class. The other students were directed to treat that expertise as valid, by listening, taking notes, and asking questions. Knowledge was about doing, instead of something that existed as correct or incorrect. The teachers actively encouraged students to critique and question the school curriculum and other sources of authority. In fact, Ukpokodu (2011) found that a major theme in mathematics’ instructors culturally relevant teaching practices was deconstructing the idea that mathematics was a culturally neutral subject that held universal truth. This deconstruction directly conflicts with beliefs that mathematics is culture-free or culture-neutral.

Ladson-Billings (2006) intentionally did not say exactly how to enact CRP. She explained that even if she could tell instructors how to enact it, she would not want to. She said that teachers would likely do what was recommended with good intentions, but without deep, critical thought about the individual students in the classroom—which is key to teaching in a way that recognizes students’ own cultures. Still, other researchers have taken up the framework of CRP and further outlined how it could manifest in practice. In a literature review of 45 articles on CRP, Morrison et al. (2008) outlined themes of how instructors supported their students for each of the three tenets of CRP. For academic success, instructors held high academic expectations by modeling and clarifying the content, planning activities so students had positive first encounters with the material that played to their strengths, taking personal responsibility for students’ success, creating nurturing environments where students felt a sense of belonging, and making behavioral expectations explicit. Morrison et al. (2008) said that the research they reviewed had the limitation of incorporating CRP with homogeneous classrooms, e.g., classes with all African-American or all Latino/a students. Thus, when the instructors said high academic expectations, they meant for all the historically marginalized students in their class.

In more heterogeneous classrooms, we interpret practices towards academic success as being culturally relevant only if they are intended to support students who have faced structural inequities. As student populations become more and more diverse, enacting CRP depends on how instructors perceive students’ cultural identities. Identity literature has tended to focus on the identities of students or the identities of instructors (Darragh, 2016; Graven & Heyd-Metzuyanim, 2019; Pozzer & Jackson, 2015), without much focus on how instructors perceive students’ identities. Still, literature reviews highlight some general ways of perceiving identity that could apply to instructors perceptions of students. Pugach et al. (2019) conducted a review of empirical teacher education research and found that researchers tend to conceptualize teacher candidates’ identities as unidimensional rather than intersectional. Pozzer and Jackson (2015) found a prominent conceptual distinction about identity in the literature was whether researchers were treating student identity as static (something they carry) or dynamic (something continually negotiated). These distinctions add complexity to enacting CRP, as understanding students’ identities and cultures might not be straightforward.

For cultural competence, instructors reshaped the prescribed curriculum to integrate non-mainstream content into the Eurocentric curriculum, built on students’ knowledge and lived experiences, and encouraged relationships between the institution and the community. To foster sociopolitical consciousness, instructors employed a critical stance toward the literature being consumed, engaged students in social justice work, made explicit the power dynamics of mainstream society, and shared power with their students in the classroom.

### Culturally relevant pedagogy in STEM

Many examples exist of how these teaching strategies have been made compatible with STEM content. Indeed, Ladson-Billings (1995) based her theory of CRP on the work of teachers of mathematics. Groups such as Science Education for New Civic Engagement and Responsibilities (SENCER) have emerged to enable college faculty to center their science curriculums around complex examples of civic engagement (Middlecamp et al., 2006). Various education researchers have demonstrated how instructors can adopt CRP in STEM given their own students’ contexts. This section gives concrete examples of how each tenet could be or has been enacted with some STEM content, starting with academic success.

In the college context, Johnson and Elliot (2020) said instructors in mathematics and science departments could enact the tenet of academic success by recognizing the distinction between preparation and aptitude for STEM (Johnson & Elliot, 2020)—providing students with pathways for success regardless of previous preparation. This could involve using active learning and group work strategically. Relevant to the HSI context, Johnson (2011) showed how two middle-school teachers participated in professional development and adopted CRP into their daily science instruction, which resulted in benefits for their Hispanic students. It involved setting students up for academic success by incorporating literacy and language strategies, creating a positive classroom climate in terms of participation, maintaining high-expectations for success, and fostering relationships between teachers and students. As part of the language strategies, teachers took a conversational Spanish course and began using words and phrases with students. The teachers also incorporated cultural competence by tying science concepts to relevant issues including agriculture and transportation.

For the second tenet of cultural competence, science instructors have worked with indigenous communities to show how the epistemologies of Western and Indigenous science are not incompatible, but can be woven together (Bang & Medin, 2010). The instructors designed a science curriculum with Native community members that centered on ecosystems and the idea that humans, animals, and plants are all related. The curriculum showed students that science involved practices that were relevant to their tribes rather than something alien. Relevant to the undergraduate context, researchers have shown how biology instructors have infused their teaching with culturally relevant, historical, social context (Chamany et al., 2008) and incorporated lab opportunities that allowed students to test samples from their respective towns (Siritunga et al., 2011). Finally, Johnson and Elliot (2020) suggested that instructors in mathematics and science departments can adhere to this tenet by instructing in a way that makes students feel like they belong with their current identities. They suggested that instructors can do this by countering the stereotype of scientists being white males that work alone and have innate abilities.

For the third tenet of sociopolitical consciousness, mathematics instructors have created lessons around social issues relevant to students’ local context (Kokka, 2019). Middle-school students used mathematics as a tool to investigate proportions of corner stores to homes in their own neighborhood in comparison to the proportions in nearby wealthy neighborhoods. Johnson and Elliot (2020) argued that, in science and mathematics departments, professors can undertake some of this work by engaging to further their own sociopolitical consciousness and understand their own biases related to race, class, gender, and their experience with their discipline. This is a prerequisite to teaching students sociopolitical consciousness because instructors need to understand their own biases before being able to reveal them to their students.

### Inconsistencies between espoused beliefs and practice

While beliefs have been found to influence practice, studies have also found inconsistencies between espoused beliefs and practices. Moreover, scholars have found that instructors could express differing beliefs about their discipline as a field of study and discipline as a school subject (for mathematics, Beswick, 2012; for science, Kang & Wallace, 2004). Thus, even if instructors expressed rigid epistemological beliefs about the knowledge within their disciplines being free of culture or culture-neutral, they might still be willing to follow practices that align with CRP. For example, Cohen and Ball (1990) showed how instructors were able to enact new teaching policies that emphasized student mathematical understanding despite holding on to old beliefs that justified the policies that emphasized computational skills. Ball and Cohen wrote, “They appeared not to notice any contradictions between the two sets of policies, and seemed entirely untroubled by their juxtaposition. Teachers spoke and acted as though the two were entirely compatible” (p. 334). The teachers took the new policies and found a way to incorporate them in a way that aligned with their original beliefs.

While individual beliefs play an important role in guiding instructor decisions, beliefs are part of a larger system that guides instructor decision-making (Nespor, 1987; Schoenfeld, 2010). Schoenfeld (2010) explained that the belief systems of instructors and the ability to implement corresponding practices are slow to change and frequently co-develop over time. An important factor in these systems is the social contexts that the instructors work within (Ernest, 1989; Raymond, 1997). Hoyles (1992) observed that teachers’ actions and beliefs were more influenced by social factors and classroom conditions rather than cognitive structures and beliefs of the individual teachers. To understand the relationship between beliefs and pedagogical practice as part of a larger system that leads to instructor decision-making, we consider the framework of practical rationality.

## Theoretical framework

**Fig. 1:**
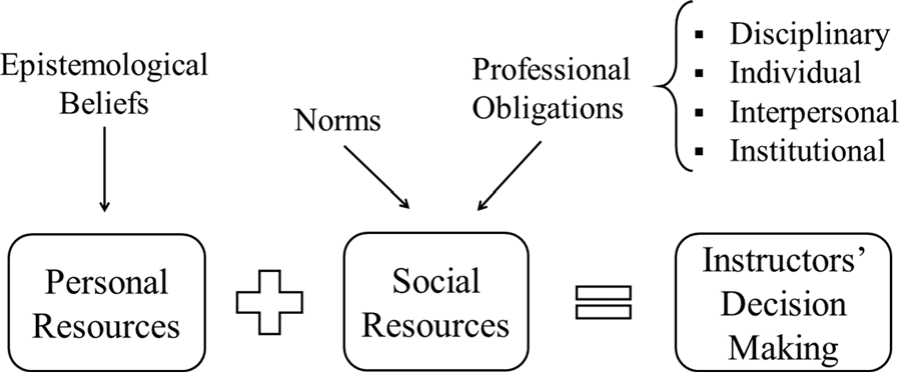
Diagram of contributors to instructional decision-making in the Theory of Practical Rationality (Chazan et al., 2016; Herbst & Chazan, 2012)

The theory of practical rationality^1^ (Herbst & Chazan, 2003, 2012) incorporates both instructors’ personal resources such as beliefs about knowledge or learning and social resources to explain instructional decisions (shown in Fig. 1). We chose this theory because it allows us to connect how epistemological beliefs (a personal resource) and commitments to different social groups (social resources) impact decisions to enact tenets of CRP. This theory takes into account that instruction is situated in interactions between the students, teachers, content, and environment (Cohen et al., 2003). When instructors enter the classroom, they enter into a social relationship guided by the didactical contract (Brousseau, 1997; Herbst, 2003). The didactical contract is a set of norms, usually tacitly expected, that guide the interactions between the instructor and students surrounding the joint construction of knowledge. For example, norms around the division of labor—who habitually is responsible for what—are part of the didactical contract (Brousseau & Warfield, 1999; Herbst & Miyakawa, 2008). In undergraduate classrooms, instructors are usually expected to propose the appropriate tasks that will help students understand the content and the students are expected to carry them out. Practical rationality is applicable to this study because incorporating aspects of CRP is often a departure from instructional norms of college undergraduate science and mathematics courses. This theory offers rational justifications for why instructors might choose to depart from norms of the classroom prescribed by the didactical contract.

The theory of practical rationality posits that four professional obligations provide justifications for departing from normative decision-making: (1) The disciplinary obligation, towards representing the discipline authentically or appropriately; (2) the individual obligation, towards meeting or accommodating the needs of students in the classroom as unique individuals; (3) the interpersonal obligation, towards overseeing the social dynamics within the classroom and the development of students as participants in society outside the classroom, and (4) the institutional obligation, towards upholding the policies and practices of the department or institution (Chazan et al., 2016; Herbst & Chazan, 2012). Each obligation corresponds with a community stakeholder of the classroom: the field of scientists or mathematicians, the students, the classroom and society at large, and the department and institution, respectively. These constructs are useful for this study because these communities are all stakeholders for the potential benefits of CRP enactment.

Using the conceptualization of professional obligations has helped explain discrepancies between espoused beliefs and practice. In a study of two secondary mathematics teachers, Webel and Platt (2015) found that instructors’ perceived professional obligations limited their ability to realize their expressed goals. Both the individual and disciplinary obligations acted as constraints on what they believed would be best for their students’ learning of the content. Mesa et al. (2019) found that some of the ways undergraduate linear algebra instructors attempted to implement inquiry-based learning indicated that the mathematics tasks they gave students during class were often constrained by their institutional obligation. For example, their beliefs that students should be exposed to more opportunities to discover the content were constrained by departmental requirements on the textbook and to the amount of content that needed to be covered in a limited amount of time. The instructors managed these tensions through the worksheets they created for their students. In an empirical study of a national sample of undergraduate mathematics instructors, Shultz (2020) found that learner-focused beliefs often predict the use of inquiry-oriented instructional practices, but recognition of the interpersonal and disciplinary obligations (depending on which inquiry-oriented practice was at stake) can work in opposition of those beliefs. And, conversely, the individual obligation predicted more use of inquiry-oriented practices than beliefs alone would indicate.

There is little research about the beliefs and professional obligations of instructors who practice CRP. In a case study of a middle-school science teacher, Kelly-Jackson and Jackson (2011) described the teacher not just as practicing CRP, but as a culturally relevant teacher due to the types of beliefs and ideologies that guided her practice. This included her epistemology that knowledge is continuously recreated, and that students are participants in the construction of knowledge. Not much is known about the relationship between STEM instructors’ beliefs and their enactment of CRP beyond conjecture (e.g., Johnson & Atwater, 2014).

It remains an open question whether instructors who do not express these beliefs can still practice CRP in a meaningful way. There is preliminary evidence that they might not be able to. Robertson and Elliot (2020) found that novice physics instructors showed a hesitance to teach responsively, i.e., building on students’ beginning ideas without evaluation. An instructor cited concerns that they would risk damaging what students took to be the objective, impartial truth (Robertson & Elliot, 2020). An instructor wrote in his teaching philosophy that “an emphasis on the mechanism of thinking without holding the conclusion’s truth value to a nonnegotiable standard is absurd” (Roberson & Elliot, 2020, p. 746). Yet there is already evidence in other contexts that beliefs and practices do not always align, but professional obligations can explain the rationality behind the misalignment.

For decisions to use CRP, we suspected that the framework of practical rationality would be most detectable in the data through the individual obligation. The individual obligation plays an especially crucial role because CRP is often a choice to customize pedagogy to a students’ individual cultural identity. Ladson-Billings (2006) urged instructors that enactment of CRP was dependent on their attention to students’ unique individual needs. How instructors think about student identity and how that materializes in their decisions to recognize the individual obligation is tied to enactments of CRP (Ladson-Billings, 2006).

### Research questions

Given that some STEM instructors express epistemologies that are seemingly incompatible with CRP, and given the urgent needs of increasingly diverse undergraduate student bodies entering STEM fields at HSIs, we ask the following questions:

1. How are STEM instructors at HSIs enacting culturally relevant pedagogy, if at all?
2. Are STEM instructors at HSIs who claim their discipline is culture-free still open to practicing culturally relevant pedagogy?
3. Can the dissonance between instructors’ epistemological beliefs and decisions to use culturally relevant pedagogy be explained by the ways instructors recognize professional obligations at HSIs?

## Methods

### Sample

We used a random stratified sampling technique based on the 2018 Carnegie Classification public data to identify the institutions we contacted to participate in this study. We sent an email invitation to department chairs or secretaries to forward to their faculty. This paper is based on 40 semi-structured interviews with undergraduate STEM instructors from 27 different HSIs. We aimed to explore a wide variety of HSI contexts. Participants represent a range of STEM disciplines (17 from biology, 7 from chemistry, 6 from physics, and 10 from mathematics), institution categories (11 associate’s, 5 bachelors', 8 master’s, 16 doctoral) and locations (20 Southwest, 10 West, 3 Midwest, 2 Southeast, 2 East, 3 Puerto Rico). Participants described their social identifiers for gender (women = 15, men = 25) and race (Asian = 2, Caucasian = 5, Hispanic = 5, Indian = 1, Latina = 1, Latin White = 1, Puerto Rican = 1, South Korean = 1, Turkish = 1, White = 22)^2^. Participants held a range of positions (29 tenure or tenure-track, 11 non-tenure track instructors). Three of the mathematics faculty and one of the chemistry faculty had an academic background in education research, which we mention in case there is a difference between their perspectives as compared to faculty who have backgrounds solely in their own disciplines.

### Interview protocol

The interview protocol was created and edited by the STEM Equity Project (www.stemequity.net). The protocol was piloted eight times by three of the authors with colleagues that taught in all four targeted disciplines (biology, chemistry, physics, and mathematics). All 40 interviews were conducted and recorded by the first author over the Zoom platform and lasted for an hour. Interviews were transcribed using the automated service otter.ai and by the service www.rev.com when the automated transcription seemed inaccurate. Participants orally consented to participate and be recorded after hearing a statement about the interview content which warned them of potential discomfort due to talking about barriers they have faced in their careers. Texas State University’s Institutional Review Board granted approval for all research activities involving human subjects in this project (IRB # 6838).

The participants were never directly asked whether they used CRP. Nor were they asked if their discipline was culture-free or to describe the nature of their discipline. Leaving questions purposefully vague was intentional. We were interested in how instructors at HSIs were making sense of instructing a large percentage of Latinx and Hispanic students, so we did not want to ask leading questions that would distort how these student populations were being served. We suspect that directly asking would yield more aspirational data than data that reflects classroom practice. An additional advantage of not using the terms culture-free or CRP was that instructors were able to explain the reasons behind their teaching practice with their own vocabulary. Thereby we were able to study instructors’ espoused beliefs with a shared vocabulary between the researcher and participant, as stressed by Speer (2005). A necessary limitation of this approach is that we cannot make claims about the exact number of instructors out of the entire sample that held culture-free or culture-dependent beliefs, we can only make claims about the number of participants that expressed either belief.

The findings in this study were mostly prompted by four questions from the 23-question protocol. First, participants were asked to think about a course they teach most often or have taught for the longest amount of time. Keeping that course in mind, they are asked: “Do the identities of students who enroll in that course influence your approach or the way you teach it? If so, how?” Second, they are asked, “How would you describe the culture or climate for students in your department in terms of supporting their identities?” Third, they are asked if “needing to address or accommodate the needs of students” or “concern for maintaining a good or inclusive classroom environment” resonates with them as a reason for making changes in their course. Finally, they were asked: “If you’ve ever taught at another institution that is not an HSI, have you made any changes to how you teach your course since starting at your current institution?” See the Appendix for the full set of questions in the interview protocol that focus on teaching practice.

### Coding

We conducted structural coding (Saldaña, 2013) of the transcripts for instances of the three tenets of CRP (academic success, cultural competence, and sociopolitical consciousness), comments that reflected the epistemology that their field was culture-free, and evidence that identity did or did not play a role in their practice. While identity is strongly linked to background and culture, we tracked identity separately due to the interesting and seemingly contradictory ways we heard instructors talking about identity. Our codebook in simplified form is shown in Table 1. Coding was conducted electronically using MAXQDA.

**Table 1:** Code names, descriptions, and examples

Code	Description	Example
Academic success	Expresses the intent or willingness to take into account students’ backgrounds, cultures or identity to set them up for academic success	“Definitely gender does. I see a lot of women in science who either have imposter syndrome or are not confident in their abilities. And I think that they're less likely to come and ask for questions. So I try to push them towards that.”
Cultural competence	Expresses the intent or willingness to set students up to understand things relevant to their own or other students’ cultures	“Because all the pictures were just sort of tan. […] When I'm doing skin are the ones that have lots of tattoos, or lots more piercings than here in western [state]. Some of my students have never traveled out of this area of [city], and sometimes those big earrings that kind of stretch the ear, but it's not a common sight that we see out here. And so trying to show them pictures of what people look like in other areas.”
Sociopolitical consciousness	Expresses the intent or willingness to help students use the discipline to develop the skills to critically “identify, analyze, and solve real-world problems” that tie to sociopolitical issues that directly impact the students being taught This can include bringing up issues that are controversial	[Only one example, see Results section.]
Culture of no culture	Evidence that they believe that culture is somehow absent or neutral in their discipline	“So it doesn't matter where you were born, you know, the theory of evolution is still the same way.”
Identity: YES	There exists evidence that they intend or would like to take into account student identity in their teaching practice	“We have very high first-generation population. About a about a third or Hispanic […] So largely how those different student identities affect my teaching is for awareness in the barriers that some of them have, especially this semester [during the pandemic], some of them have home situations where it's really hard to focus on their work. […] I have a flexible extension policy because I know that sometimes they have to go to work.”
Identity: NO	Considers student identity as not playing a role in their practice	“No, it does not. I do have all kinds of the students. This time I do have a majority of the students,[…] most of them are from minority groups. It does not matter. It does not influence me.”

The first author coded the full set of interviews. The second, third, and fourth authors acted as second coders to establish interrater reliability on all six codes. The unit of analysis was an answer to a question. Coders established reliability by independently coding responses to two questions from twenty randomly selected interviews (50% of all interviews). Most of the relevant data emerged from those two questions. We report the kappa coefficient (Cohen, 1960) to not misrepresent the extent of agreement based on the absence of codes occurring.

Every instance of a culture of no culture code or a sociopolitical consciousness code, even if not randomly selected for interrater reliability, was later reconciled by at least two authors due to their low frequency. For Cohen’s kappa coefficient, agreement is considered fair for ranges 0.21–0.40, moderate for 0.41–0.60, substantial for 0.61–0.80, and almost perfect for 0.81–1.00 (Landis & Koch, 1977). Our agreement and kappa coefficient for each code is shown in Table 2. The kappa score for cultural competence was low due to the rarity of instances in the data, but our percent agreement was high (93.3%). The kappa score for sociopolitical competence was undefined because we found no occurrences of this in randomly selected transcripts we coded. All other agreements had kappa scores of 0.42 or better.

**Table 2:** Percentage agreement and kappa scores to establish interrater reliability (Cohen, 1960)

Code	% Agreement	κ
Academic success	82.2	0.63
Cultural competence	93.3	0.37
Sociopolitical consciousness	100	Undefined
Culture of no culture	93.5	0.66
Identity: YES	50.2	0.42
Identity: NO	84.4	0.61

### Positionality

The interpretation of participants’ data through theoretical framing and coding was influenced by the authors and their own cultural backgrounds. We thus include a brief positionality statement for each author. For all of us, earning STEM graduate degrees both required privilege and has afforded us additional privilege; these experiences have also informed us about the nature of the STEM communities’ commonly held beliefs that they are culture-free.

Mollee Shultz’s positionality statement: I identify as a Chinese-American, cisgendered, heterosexual woman. I was adopted and grew up in a White household, limiting my first-hand experiences of systemic inequities in education. I hold an M.S. in mathematics and a Ph.D. in mathematics education. I have worked as an adjunct faculty teaching mathematics at three 2-year HSIs. Having experienced the adjunct life of teaching many courses and students, long commutes, and no healthcare, I understand that asking instructors to teach with CRP to each individual student is a high demand. This makes me interested to hear how instructors express enacting it and what could make it more feasible.

Jayson Nissen’s positionality statement: Identifying as a White, cisgendered, heterosexual man provides me with opportunities denied to others in American society and science and limits my perspectives on inequities in science education. My experience growing up poor and serving in the all-male submarine service motivated me to reflect on and work to dismantle my privilege and oppressive power structures in science. I was trained in physics with no explicit instruction in the philosophy of science, and I was motivated in this work to better understand the relationships between philosophy, inequity, and instruction in STEM disciplines.

Eleanor Close’s positionality statement: I identify as a White, cisgendered, heterosexual woman. I hold B.A. and M.S. degrees in physics and an Ed.D. in Curriculum and Instruction. I am currently an associate professor of physics at a regional HSI; previously I taught for 8 years at a small liberal arts university and for 3 years as a high school science teacher. As Director of the Physics Learning Assistant program at my institution, I have had the privilege of participating in deep conversations about instructional experiences with both faculty and undergraduate students, and supporting instructional change efforts across multiple STEM departments. These experiences have motivated me to understand the structures, beliefs, and relationships that shape STEM instruction and classroom interactions at HSIs.

Ben Van Dusen’s positionality statement: I identify as a White, cisgendered, heterosexual man. These identities are aligned with common U.S. beliefs about a “normal” physicist, further enabling me to ignore the role of culture in the science community. I am currently an assistant professor at a research-intensive institution, but I worked for 5 years at a regional HSI and 5 years as a high school science teacher. My experiences at the HSI teaching physics courses to future elementary teachers and supporting my colleagues in transforming their STEM courses using Learning Assistants (Barrasso & Spilios, 2021) informed my perspective on this project. They provided me insight into the deep care that many HSI instructors have for their students and the structural barriers that often prevent them from supporting their diverse students in the holistic ways that they want.

## Results

The results are organized in three sections. The first section describes how the three tenets of CRP manifested in instructors’ expressed practice. This gives a tangible understanding of what CRP can be when we report that instructors are deciding to enact it. The second section illustrates how culture-free epistemological beliefs and CRP arose in our sample. Three cases illustrate the ways those beliefs and practices can interplay, mapping these instructors’ justifications onto the framework of practical rationality. The third section focuses on practical rationality, describing how the individual obligation, in the form of accounting for student identity, plays a role in decisions to use or not use CRP.

### Manifestations of culturally relevant pedagogy: “Not just a parade of dead white guys”

Manifestations of CRP were described by 90% (*n* = 36) of instructors. The number of interviewees per discipline and total number of interviewees that mentioned a practice coded as one of the three CRP tenets are shown in Table 3.

**Table 3:** Percentage and number (in parentheses) of participants by discipline that expressed enacting or willingness to enact CRP

Evidence of	Biology (*n* = 17)	Chemistry (*n* = 7)	Physics (*n* = 6)	Mathematics (*n* = 10)	Total (*n* = 40)
Academic success	94% (16)	86% (6)	83% (5)	80% (8)	75% (35)
Cultural competence	29% (5)	14% (1)	17% (1)	20% (2)	23% (9)
Sociopolitical consciousness	0%	0%	0%	10% (1)	3% (1)
No apparent enactment of any culturally relevant pedagogy	6% (1)	14% (1)	17% (1)	10% (1)	10% (4)

Columns can sum to more than *n* because participants often expressed more than one tenet of CRP

Academic success related to CRP was initially a challenging code to identify reliably due to the many moves that instructors related to ensure the academic success of their students. We distinguished these enactments as part of CRP when instructors remarked that they did them in response to students’ perceived backgrounds, cultures, and identities; oftentimes instructors related these to structural inequalities such as not having access to good background education. That is, for every practice counted as an instance of academic success, instructors gave some indication these were practices used in order to adapt their teaching to their students’ unique needs associated with their backgrounds, identities, or cultures. For example, if an instructor talked about having a flexible extension policy, that alone would not have been a culturally relevant practice. However, if he said he had the flexible policy because the student population was one-third Hispanic and 56% “minority” and many of these students had barriers to focusing on their work, such as holding jobs in addition to school, then we did interpret it as a culturally relevant practice. The instructor from our first case study, Henry, often talked about holding high academic standards, but as a general philosophy for all students, so it did not count as an enactment of academic success. The understanding of students' context as a motivation for a practice is what made it culturally relevant.

We heard 75% (*n* = 30) of faculty describe incorporating the most common tenet, academic success. Common manifestations of this tenet included reaching out to or checking in on students individually, changing due dates to accommodate students’ schedules, taking extra time to ensure students had the background knowledge they needed to succeed with the current material, keeping expectations around content understanding high, and getting students the resources they needed (e.g., textbooks, calculators, internet). Specifically relevant to the HSI-context, some instructors talked about speaking Spanish or directing students to another instructor or tutor who did.

The CRP tenets of cultural competence and sociopolitical consciousness were less common. We heard 22% (*n* = 9) of participants relate practices that fit the description of cultural competence. The most common example of the theme, shared by four instructors, was including diverse representations of historical leaders of the field. A physics instructor said, “I try to make sure it’s not just a parade of dead white guys.” An example of how an instructor drew from students’ cultural experiences included using medical examples the students brought in from their own lives or pop culture to illustrate anatomy issues. Specific to the HSI context, instructors expressed making connections from the vocabulary of the discipline to Spanish and focusing on illnesses relevant to the local low-income Hispanic population. Some ways instructors educated students on others’ experiences were through showing pictures of different potential patients (with a wider variety of skin tones, piercings, and tattoos than students were familiar with in their communities), or contrasting foods/plants or food regulations to other places.

We only coded one instance of an instructor using sociopolitical consciousness. The instructor, a mathematics educator, included an article on special education in a mathematics for future teachers course. He included it in response to students’ misconceptions about students in special education. We considered it an example of sociopolitical consciousness because the way exceptional students are perceived and treated in society is an issue of equity. Educating future teachers to question their perceptions about special education furthers their sociopolitical consciousness.

We saw a clear correlation between instructors who expressed enacting CRP and those that recognized the individual obligation, especially in the form of student identity playing a role in their teaching. Table 4 shows the percentage of instructors who reported students’ identity playing a role in their teaching as a percentage of the instructors who expressed enacting each of the three tenets. To enact cultural competence and sociopolitical consciousness, instructors tend to have to think about the identities of their students; or, in the case of the mathematics educator, the identities of the future students of their students. This obligation to the individual student seems to be a part of the decision-making of instructors who choose to use CRP. For the tenet of academic success, the correlation with identity: yes is high but not exact because we defined academic success to not be solely about whether the instructor takes extra action to support students on account of identity, but also on account of other structural inequalities often related to students’ backgrounds or cultures (e.g., poor academic preparation, economic circumstances, general categories like “underrepresented”). These all had the commonality of meeting individual needs, i.e., still recognizing the individual obligation in their decision-making, but did not have to specifically recognize the students’ particular identities like the other two tenets.

**Table 4:** Percentage and number (in parentheses) of instructors who expressed student identity played a role in their teaching, out of the instructors who expressed enacting each of the three tenets

	Academic success (*n* = 35)	Cultural competence (*n* = 9)	Sociopolitical consciousness (*n* = 1)
Evidence or explicit statement that student identity plays a role in teaching	86% (30)	100% (9)	100% (1)

### Culture-free epistemologies: “Mathematics has no color”

We found evidence that 18% (*n* = 7) of faculty expressed culture-free epistemological beliefs across the four STEM fields we investigated (see Table 5). They professed these beliefs without being directly asked. As shown from the literature review, epistemological beliefs are closely related to beliefs about teaching (Beswick, 2012; Cross, 2009; Ernest, 1989; Johnson, 2011; Martínez-Sierra et al., 2020), so we investigated the prevalence of CRP use by instructors who professed culture-free disciplinary beliefs. As shown in Table 5, many instructors who professed culture-free epistemological beliefs also considered student identity (5 of 7) and at least one tenet of CRP (6 of 7) in their teaching.

**Table 5:** Percentage (and number) of participants that expressed culture-free beliefs and also expressed consideration of student identity or enactment of CRP in their teaching

Evidence of	Biology (*n* = 17)	Chemistry (*n* = 7)	Physics (*n* = 6)	Mathematics (*n* = 10)	Total (*n* = 40)
Culture-free epistemological beliefs concerning their discipline	12% (2)	14% (1)	17% (1)	30% (3)*	18% (7)
Culture-free epistemological beliefs AND identity: yes	12% (2)	14% (1)	17% (1)	10% (1)	13% (5)
Culture-free epistemological beliefs AND enactment of CRP	12% (2)	0%	17% (1)	30% (3)	15% (6)

*One of the mathematics instructors was speaking about the beliefs of instructors in her department, not her own

**Fig. 2:**
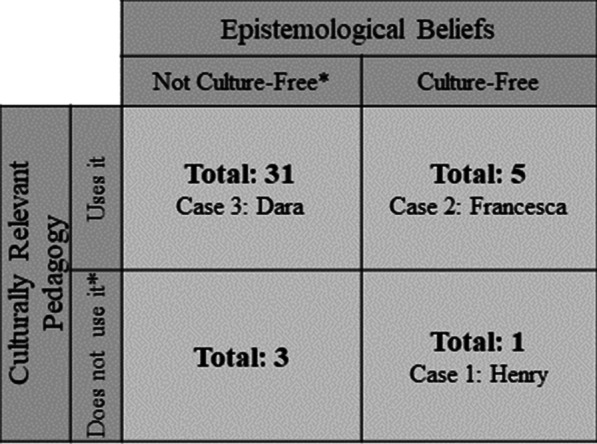
How the three case studies cover a combination of epistemological beliefs and CRP usage. *We report that participants did not express using CRP and did not express culture-free beliefs. This is distinct from reporting that participants did not enact CRP and did not hold culture-free beliefs

We report three in-depth cases of how professional obligations play a role in determining how instructors make decisions to use CRP given their beliefs, chosen for their unique combinations of CRP use and epistemological beliefs (see Fig. 2). Their information is shown in Table 6. We do not report a case from the combination of not using CRP and not holding culture-free epistemological beliefs because we did not know enough about those instructors. While they did not profess using CRP nor holding culture-free epistemological beliefs, they also were not explicitly against CRP nor did they express culture-dependent beliefs. It was therefore difficult to come to concrete conclusions about what was happening and why, in terms of CRP enactment.

**Table 6:** Demographics, position, field, and institution type and location for three case studies

Pseudonym	Social identifier	Position	Carnegie classification	State	Field
Henry Danielyan	White man	Department Chair	Associate's Colleges: High Transfer-Mixed Traditional/Nontraditional	CA	Chemistry
Francesca Sullivan	White woman	Adjunct Professor	Associate's Colleges: High Transfer-Mixed Traditional/Nontraditional	CA	Mathematics
Dana Gilbert	White woman	Assistant Professor	Associate's Colleges: High Transfer-Mixed Traditional/Nontraditional	TX	Biology

#### Case 1: culture-free disciplinary epistemology and culture-free pedagogy

Of the six instructors who expressed culture-free disciplinary epistemologies, only one showed no interest in using CRP. Henry, a chemistry professor, was adamant that the best teaching would ignore students’ cultures. He did not express enacting CRP on account of rationales related to the individual obligation. When asked if the identities of students influence his approach to teaching, he responded, “no, not at all. […] A lot of times, I don’t realize what their names are or who they are.” He continued,

You want to stop racism, stop talking about it. [...] People talk about [race] all the time, they’re used to it, and it hurts people. When I teach a student, I don’t care what their color is or what their gender is, none of that matters.

He reflected a color-evasive racial ideology—that non-recognition of race is preferable and inclusive.^3^ He explained that the approach to teaching that does not consider students’ identity was best because it respects their individuality by not placing them in a group. He stated that “humans are humans”.

Henry expressed care for the interpersonal environment of his classroom, but his evidence that his classroom was inclusive was built on his own perceptions. He claimed that people were not afraid to “speak out because of their identity, color, or gender” because he has “never noticed anybody being uncomfortable.” He said that people were “pretty loud” in his class, and said that the environment itself has never felt stifling. If students were not talking, he believed it was because they were shy and those students would be just as quiet in any other classroom. He felt that his approach of treating each person as a person without regard for their culture was the best way to foster an inclusive environment. Henry was on the far end of the spectrum in terms of his explicit stance against epistemological beliefs that align with CRP and his rejection of the relevance of student identity. However, the themes of not wanting to make assumptions and of judging the inclusivity of the classroom by the lack of noticeable conflict were themes that emerged beyond this case.

From the lens of practical rationality, Henry did not profess a strong individual obligation and instead felt students should be responsible for their own success. He said that “it’s a sink or swim situation.” Oftentimes college instructors recognize the individual obligation to a lesser degree than their K-12 counterparts (Ko et al., 2021), possibly due to seeing students as adults responsible for themselves. Henry felt his students’ success was outside his own power. He did a review in the first couple weeks of the semester and told his students that “It’s your time to catch up. And if you can catch up, you’ll be in very good shape for the rest of the semester.” He used a challenging text and wrote exams “very much what you’d be used to at the higher institutions, the more elite institutions, four or five questions, many concepts in one, time limits”, perhaps to meet standards he associated with the discipline.

By placing individual responsibility on students to succeed at the way physics is taught at elite institutions, Henry saw students and their cultures from a deficit perspective rather than something that could be leveraged to learn the material in new ways. He said, “You can't change who people are or their cultures, it's got to happen organically. If you want to do it in school, well, then you got to care”, implying that students may not do well because their culture predisposes them to not care about learning chemistry.

This approach to instruction of maintaining high academic standards without taking personal responsibility for meeting his students’ individual needs, alongside culture-free beliefs about teaching and learning chemistry, demonstrates a perfect recipe for an instructor who would not see the importance of enacting CRP. The next case features an instructor who similarly held culture-free disciplinary beliefs and high academic standards but differed on one key feature: she recognized the individual obligation by taking personal responsibility for each student’s success.

#### Case 2: holding a culture-free disciplinary epistemology while using culturally relevant pedagogy

Francesca expressed culture-free beliefs about her discipline, yet expressed that she incorporated or was willing to incorporate aspects of CRP. Of the six instructors with culture-free disciplinary epistemologies, five expressed that they used or were willing to incorporate practices that fell under academic success and one instructor expressed that she used a practice that fell under the cultural competence tenet of CRP. Francesca was an adjunct professor at a 2-year HSI in California who also had experience teaching in other environments (“prep schools and inner-city schools”) and countries (Europe, Mongolia, China, Japan, and France). She expressed her beliefs about mathematics as follows: “I've taught in all sorts of environments and the broad statement I'd like to make is that I find students to be extremely similar. Maybe it has to do with mathematics.” When asked about the culture in her department, she stated,

We don't see a color or sex or anything. We see a raw being that you can teach mathematics. To me, mathematics has no color. Maybe that's debated, but you look at something like the ocean and the color currents in it. And you know that those are all governed by mathematics. And many forces. And that's just nothing about ethnicity in it. It's bigger than that.

Her self-reported beliefs show an epistemological stance that mathematics has some truth that is beyond culture.

Yet, Francesca showed willingness to incorporate CRP on account of practical rationality’s individual obligation to the students. The form of her CRP was that of academic success, maintaining high standards on students’ understanding of the mathematics content and taking personal responsibility for their success (as in Morrison et al., 2008). While not acknowledging race as a reason for these practices, she talked about enacting these in terms of systemic issues of (lack of) student preparation and addressing her students’ needs given the school systems they came from. Speaking in the context of some of the places she has taught with diverse student backgrounds, she took responsibility for helping students attain a genuine understanding of the content regardless of what prior mathematics educational opportunities they had, because she said students are not satisfied with merely getting good grades. Good grades had to be coupled with comprehension of the content, otherwise they would be frustrated. She further said that though students were often initially shocked at the difficulty of her class, which she compared to drinking from a firehose, her students learned that she is there to help them through it.

The second way she emphasized the importance of CRP on account of the individual obligation was through her appreciation of students’ unique ways of thinking. While she did not believe mathematics was culturally dependent, she believed that the way students understood mathematics is. She said,

I find that different ethnicities have different minds. They’re all very good at mathematics, but it’s like they have flavors to them. And this is what I find so exciting about mathematics. Because mathematics has an infinite number of sides to it, like a diamond, and the way people understand is different. And it’s so motivating. [...] If you are into mathematics, you know 10 different ways to explain every single thing. When a student doesn’t understand what you’ve said, it’s an opportunity to rejoice with that student and then try another way.

She appreciated that students understand things in distinctive ways and took personal responsibility for helping find what way would be effective for each of her students. She listened to their individual difficulties and would come back to the next class prepared to address them. She claimed that therefore only mathematicians should teach mathematics, seemingly because of their deeper content knowledge and ability to approach the subject multiple ways. In addition to listening to students’ individual difficulties, she also valued the group as a whole.

From the perspective of practical rationality, Francesca created a class that valued academic success in part on account of the interpersonal obligation. Instead of dropping her academic standards to accommodate students, she wanted to create a social environment where everyone could succeed together. She said she tried to create an “esprit de corps.” She stated, “you don’t accommodate individuals, but rather, they grow into a team.” It was important to her that her class was inclusive, and that “everybody’s on the same page, even though everybody’s different.” She wanted to make it possible for anyone in her class to understand the content, even or especially if they came from an academic background with unhelpful mathematics instruction in the past. She said, “I ensure them that this is definitely not their fault. I don’t even care if they’ve been bad, rebellious or didn’t listen, or whatever they’re accused of. It’s not their fault.” She entered the relationship with the assumption that all students had the capacity to learn and just had not been exposed to the best instruction or learning environment yet. Although she may have reflected a deficit mindset or conversed with other teachers who had a deficit mindset, she saw it as her responsibility to create a course where students could all understand the content regardless of their past mathematics preparation. The way she taught with high academic standards and a value on effort, both from herself and her students, was in part due to this commitment to an inclusive interpersonal environment.

#### Case 3: did not express a culture-free disciplinary epistemology and used culturally relevant pedagogy

Of the 35 instructors who did not give any explicit indication that they believed their discipline was culture-free, 31 instructors expressed enactment or willingness to enact elements of CRP. Dana Gilbert, a biology professor, is an example of instructors in this category. She did not explicitly say she believed biology was culturally dependent (we never asked), but the way she taught implied that certain knowledge associated with the discipline would be more useful depending on culture. She implemented two tenets of CRP. The first was cultural competence, which she implemented by teaching her students about culturally relevant illnesses. Her students were often nursing majors. She explained, “I have a tendency to stress the illnesses and the disorders that are particularly going to affect our population, being a low-income Hispanic population that I teach to.” She emphasized certain illnesses that are more prevalent in her region such as diabetes, and she incorporated them into her course through assigned projects. From the standpoint of practical rationality, these practices recognized the individual obligation by informing students about things that might be useful to them as individuals going into the workforce in their region, and recognized the interpersonal obligation by thinking about how the preparation of future nurses would impact the local society.

More generally, she incorporated cultural aspects of her discipline by asking students to bring current examples from popular culture. She tells students, “If a famous person, sports hero, movie star, someone that everybody has heard of has an injury or an illness, bring it up in class.” She wanted students to understand human anatomy and physiology in a way that they could understand local news stories or understand, if a family member sees a doctor, what the diagnosis for the injury or illness means. Relevant to the time period when the interviews were conducted, she said “We’ve talked a lot about COVID-19 in the last nine months.” All of these instructional practices were consistent with her professed goal of having students be informed citizens (an aspect of the interpersonal obligation) and understand how biology applies to their individual experiences (an aspect of the individual obligation).

Secondly, Dana incorporated academic success by setting up her course in a way that accounted for students’ individual identities outside of the classroom. She remarked that her students were not traditional students, but instead are older, working, and have children. They are “ones who possibly have waited until their kids are in school, and now they’ve gone back.” This affected her teaching by changing when she has key due dates. She usually places due dates on Friday or Saturday evenings because, “students consider Sunday to be time when they’re going to spend with their family” and early in the week students are often busy with their own work. Her concern for her students as whole persons with competing priorities was evidence of the individual obligation playing a role in her practice.

### CRP and the individual obligation to students’ identity

From the standpoint of practical rationality, many instructors chose to use CRP based on attending to the individual obligation, which included considering how to best support students with their respective identities. The way instructors perceived their students’ identities provided an illustration of what instructors perceive as the individual obligation. Identity is essential to understanding the enactment of CRP and thus responding to our third research question because it is often on account of students’ identities that instructors decide to implement CRP.

Note that this was not always true—instructors also expressed enacting academic success to respond to more general things about students’ backgrounds and the associated structural inequities the students faced. For example, one instructor emphasized her concern for helping students succeed through a course that tended to be gatekeeping. She also said she provided extra tutoring, and wrote a letter of recommendation for a student with health issues. These seemed culturally relevant by taking into account students’ backgrounds and helping students succeed at something that had previously kept some students out based on their backgrounds. Thus taking into account students’ identity is not automatically inferred by enacting CRP.

Instructors generally seemed attuned to some student identities, with 35 of the 40 interviewees indicating that students’ identities played a role in their instruction, as shown in Table 7. In total, 19 instructors denied identity playing a role in their instruction. Four of these instructors (e.g., Henry) were consistent in this denial. However, 15 of these 19 instructors (38% of all interviewees) related instances of students’ identities playing a role in their pedagogy even when the instructors also explicitly denied student identity playing a role in their instruction (e.g., Francesca). Other instructors echoed Henry’s color-evasive sentiments that they did not want to teach in a way that incorporated student’s identities (e.g., race, gender, sexual orientation), which came from a place of not wanting to make assumptions or stereotype their students. Another instructor said “I don’t necessarily change my teaching […] it doesn’t matter what the person looks like right?” but later described offering opportunities to make non-White, non-Asian students more comfortable to seek help.

**Table 7:** Percentage (and number) of participants that expressed or objected to student identity playing a role in their teaching

Evidence or explicit statement that student identity	Biology (*n* = 17)	Chemistry (*n* = 7)	Physics (*n* = 6)	Mathematics (*n* = 10)	Total (*n* = 40)
(1) Plays a role in teaching	100% (17)	86% (6)	83% (5)	70% (7)	88% (35)
(2) Does not play a role in teaching	47% (8)	14% (1)	66% (4)	60% (6)	48% (19)
Both (1) and (2)	47% (8)	14% (1)	50% (3)	30% (3)	38% (15)
Neither	0%	14% (1)	0%	0%	3% (1)

^a^These categories are not disjoint. Each instructor in “Both (1) and (2)” is also counted in (1) and (2)

One telling example showed how identity can play a role in teaching, but not in the way instructors expressed verbatim. Ian, a biology adjunct instructor, said that student identity did not play a role in his teaching, and that he never did anything special for Hispanic students. Yet, he also said “I actually was told something like that when I first started. They’re like, Hey, man, these kids are really high needs. You gotta bring a level of content down for them and blah, blah blah.” Ian said, “I had this impression that students have been treated that way their whole lives”, and he felt it was important to hold high academic standards for them especially because they had not been treated that way before. He recognized that future employers would hold those students to the same expectations as everyone else regardless of the challenges they had to overcome, and he wanted his class to prepare them. Thus, identity did play a role in Ian’s teaching and in how he expressed enacting academic success, just not in the way he thought the question implied.

Throughout the interviews, instructors showed resistance to using racial or ethnic markers of identity to describe how they made instructional decisions, but did use markers like socioeconomic status, 1st generation status, or other working roles like caregiver. Such markers were often used as justifications for implementing the practices associated with academic success. For example, Tiffany, a statistics instructor, said identity did not play a role in her teaching because she thinks of students “as individuals, but not necessarily […] their color, their gender or anything else.” Her expression reflected a more intersectional rather than monolithic view of students’ identity, which resulted in her denying identity playing a role in her teaching. She also said that she tried to help international students because “interpreting what’s being asked is somewhat difficult […] and it is somewhat of a disadvantage to non-native speakers.” There was something about the label of international student that was easier to use than something more politically charged like race or gender, and perhaps she did not see international as being part of identity. This implies that how instructors perceive student identity relates to what CRP tenets are enacted. The instructors who are resistant to any conversation about student race or ethnicity, for example, might be able to implement academic success by addressing other needs those groups might have. However, it does not allow instructors to directly address the cultural needs of those racial or ethnic groups.

## Discussion

We found evidence that faculty expressed culture-free epistemological beliefs in each of the four STEM fields we investigated, sometimes while simultaneously practicing CRP. Using the lens of practical rationality, we found that CRP enactment was closely related to the individual obligation, and sometimes to the interpersonal obligation. This paralleled the finding that some faculty expressed that identity did not play a role in their teaching but then also described ways in which it did. We organize this section by research questions. It is followed by implications for how departments might design the support and professional development for faculty in existing and emerging HSIs, and how future research could investigate the implementation of the two lesser-used tenets of CRP.

### Use of CRP by HSI instructors

We began by asking the research question: How are STEM instructors at HSIs instantiating culturally relevant pedagogy, if at all? Past literature has shown instances of rich enactments of CRP but has not explored if or how it is being enacted in the HSI context. As institutions are not required to incorporate any special training for faculty when they are granted HSI status, it is reasonable to not assume that instructors are incorporating CRP into their instruction. Indeed, some instructors in our study were not familiar with the acronym HSI. We found that most instructors included practices that aligned with academic success, some instructors included practices aligned with cultural competence, and only one professor included a practice that aligned with sociopolitical consciousness.

Though the sample was too small to make any general claims, the variation across fields aligned somewhat with the literature on disciplinary-specific epistemologies. The uniqueness that Mayr (2004) highlighted about the context-dependence of biology aligned with some of the reflections we heard of enactments of academic success and cultural competence by biology instructors. Though results reflected mathematics instructors were more likely to hold culture-free beliefs, aligning with the theoretical nature of mathematics as outlined by Develaki (2020), the mathematics instructors in our study did not show they were less likely to use any of the CRP tenets.

Generally, we were struck by the lack of any examples of sociopolitical consciousness, or instruction that equipped students to think critically about the fields they were studying or to think about how the tools they were learning could be applied to social issues that affected themselves or people in their community. For example, physics courses teach about energy but do not discuss how it is related to climate change. Dana did teach about diseases that disproportionately impacted the Hispanic community but did not talk about the sociological factors that shape the development of these diseases.

These findings could also be interpreted as an indication of the rich opportunities to incorporate more CRP into undergraduate STEM instruction. Instructors generally seemed caring and passionate about the success of their students and motivated to create an environment that would help students from a wide range of backgrounds understand the content. Many instructors sounded amenable to incorporating the other two tenets of CRP, but lacked the time or knowledge to do so in a meaningful way. The main explicit potential barrier we identified was instructors holding epistemological beliefs that contrasted with the way CRP situates knowledge as dynamic and socially constructed.

### Culturally relevant pedagogy despite culture-free epistemologies

Our second research question was: Are STEM instructors at HSIs who claim their discipline is culture-free still open to practicing culturally relevant pedagogy? We found that STEM instructors who claim their discipline is culture-free are still open to practicing CRP. Like Cohen and Ball (1990) found that instructors were not bothered by the juxtaposition and some were “positively eloquent” (p. 53) about how the new policies fit with the older contradictory initiatives, the instructors in our study did not seem bothered by the juxtaposition between enaction of culturally relevant practices and holding culture-free epistemological beliefs. For example, many of the ways in which Francesca explained that she supports her students correspond with CRP strategies for academic success outlined by Morrison et al. (2008). This does not discount the importance of instructors growing their own sociopolitical consciousness and thinking critically about their own epistemological beliefs. We think implementing robust forms of CRP involves challenging the neutrality of STEM knowledge. For long lasting, maximally impactful change, we agree with Henderson et al. (2011) that changing the belief systems of instructors with culture-free epistemologies would be most effective. At the same time, addressing the needs of the changing student population is urgent and must not wait for the slow process of changing belief systems.

Another thing instructors said that at first glance might conflict with the use of CRP was a self-reported unwillingness to consider students’ identity in their pedagogy. Paradoxically, recognition of the individual obligation seemed to cause instructors to deny the impact of students’ identity in some instances. We posit that while there was some color-evasiveness, as in the case of Henry, there was also prudent consideration of the individual student that guided instructors’ reluctance to report that they incorporate students’ identities (e.g., racial or gendered) into teaching. Gutiérrez (2013) noted that “essentialization, reducing a group to a single characteristic that seeks to convey the essence of that group, goes against the very idea of creating meaningful bonds with students through shared interaction” (p. 51). Some instructors perceived their students’ identities from a more intersectional view. For that reason, instructors who explicitly said they did not take students’ identities into account in their teaching still frequently showed they did take students’ identities into account in a thoughtful manner and/or used CRP.

An important caveat to explore further is whether instructors who express culture-free epistemological beliefs are willing to incorporate instruction on cultural competence and sociopolitical consciousness. While we found that some instructors expressing culture-free epistemological beliefs were interested in getting to know students’ cultures independently from the discipline, only one of the six instructors showed use of a practice related to cultural competence and none to sociopolitical consciousness. That is, we did not find much evidence that instructors expressing culture-free epistemological beliefs would be amenable to having students’ use the discipline to understand culture, much less think critically about it. This does not necessarily mean that they were unwilling to apply these other two tenets, as we never explicitly asked this.

### Professional obligations mediating disciplinary epistemologies and practice

Finally, our third research question was: Can the dissonance between instructors’ epistemological beliefs and decisions to use CRP be explained by the ways instructors recognize professional obligations? Using practical rationality to understand Francesca’s decision-making, we found that the dissonance between epistemological beliefs and use of CRP was explained by her recognition of the individual and interpersonal obligations. Thus, in at least some cases, the ways instructors recognize professional obligations can explain the dissonance between their epistemological beliefs and their instructional decisions. This finding furthers the growing evidence that professional obligations from the theory of practical rationality can help explain the relationship between beliefs and practice (e.g., Shultz, 2020; Webel & Platt, 2015).

Francesca treated students’ individual identities as dynamic in terms of how these identities interact with their understanding of the content, and expressed enacting academic success by customizing her explanations to what students needed. As part of her recognition of the interpersonal obligation, she aimed to create an inclusive classroom with an “esprit de corps”. In doing so, she incorporated academic success by maintaining high academic expectations and creating an environment where anyone can have a pathway to success. This recognition of the interpersonal obligation was inextricable with her recognition of the individual obligation.

For Francesca, there was no dissonance between believing that mathematics was culture-free while meeting students at whatever place their cultural experiences had brought their mathematical understandings thus far. What might have been more of a leap for her and other instructors with culture-free epistemologies would be leveraging students’ cultural experiences to help them understand the discipline or how the discipline could apply to real sociopolitical issues relevant to themselves or their classmates. All instructors who expressed using practices under cultural competence and sociopolitical consciousness also reported ways that students’ identities played a role in their practice. For instructors who do not use practices consistent with those tenets, learning to value how students’ identities influence the ways they interact with content might be a first step towards changing the ways they recognize their individual obligation.

## Conclusions

### Supporting instruction with CRP

The finding that instructors can practice CRP while expressing culture-free beliefs shows that changing instructors’ epistemological beliefs is not a prerequisite to instructors’ willingness to use CRP. While it is crucial for instructors to think critically about their epistemological beliefs and culture-free conceptions, such a change in orientations can take time (Schoenfeld, 2010). Given the urgent needs of students facing structural inequities in science and mathematics HSI classrooms, it is useful to recognize that instructors’ recognition of the individual and interpersonal obligations as conceptualized in the theory of practical rationality can justify instructors’ decisions to use CRP. Those motivations can be leveraged to promote CRP without first waiting for instructors’ beliefs to change. The findings from the second and third research questions give an impetus for providing resources and professional development that gives STEM instructors at HSIs ways to incorporate CRP into their existing curriculum. While not trivial, this may be a more equitable approach than waiting until instructors are convinced to change their beliefs about the nature of their discipline. Because the literature has shown that beliefs are difficult to change, it is useful to know that instructors might be willing to implement CRP without changing their core epistemological beliefs if they can be shown how it will directly benefit their students. This finding is an interesting converse of Brown et al.’s (2019) finding that instructors agreed with the theory of culturally relevant education but did not know how to implement it. Here, instructors do not necessarily agree with the beliefs behind CRP but show they already implement some aspects of it.

Facilitating change in undergraduate departments requires developing a strategy that is informed by the complex contexts in which instructors work (Henderson et al., 2011). We propose a strategy that accounts for the high demands on instructors while allowing them to meet practical rationality’s individual obligation as they have intended. STEM departments could make resources available to instructors to make it easier and more of a default option to teach responsively to their student populations. While honoring Ladson-Billings’ (2006) call to not prescribe how to teach with CRP because instructors need to think strategically about how to make content culturally relevant to their own students, departments could set up instructors to not start from scratch. Rasmussen and Ellis (2015) used the term “choice architect” to describe the work of a course coordinator in the context of undergraduate calculus courses that are taught by many instructors at a university. A choice architect is not someone who makes choices for people, but rather nudges people towards using something (in this context, perhaps a culturally relevant lesson plan or information about the sociopolitical issues relevant to the demographics of the student population) by making it readily available. For introductory STEM courses that are taught repeatedly to large numbers of students consistently from the same range of backgrounds, this could involve the creation of culturally relevant tasks that can be done collaboratively with given disciplinary content, information about the relevant social issues local students might face, and instruments (e.g., surveys) to help instructors easily collect information about their students.

### Future research

We call for two types of studies, the first focused on the instructor and the second focused on the institutional system, both related to implementing cultural competence and sociopolitical consciousness. First, this study gives cause for investigations about something we call Culturally Relevant Pedagogical Content Knowledge (CR-PCK). The cultural competence and sociopolitical consciousness tenets of CRP require a deep knowledge of both the content and the issues relevant to the student population (Brown et al., 2019; Ladson-Billings, 2014). Shulman (1986) set up the idea that there is a type of knowledge for teaching called pedagogical content knowledge (PCK) that goes beyond simply the content knowledge of a practitioner in the field. For example, Francesca suggested mathematics ought to be taught only by mathematicians because they have a deeper understanding of the content, and therefore are better prepared to explain things to students; we argue that the ability to explain a concept in multiple ways to address students’ unique ways of understanding is actually a form of PCK, and being a mathematician is not a prerequisite for developing this knowledge. Our interviews with faculty at HSIs demonstrated that some faculty are already able to make connections from their content to issues relevant to Hispanic students, as shown by the case of Dana and her biology students.

We argue that the knowledge of the content that is most relevant to students, as well as the ability to probe to find out from students how content connects with their lived experience, is a type of knowledge (CR-PCK). The context-dependent nature of who the students are, what the content area is, and what the instructor decides is culturally relevant will make measuring this construct challenging; less straightforward than Hill et al. (2005) with their assessments of mathematical knowledge for teaching. However, giving CR-PCK a label, and investigating how instructors acquire it and how students are impacted by instruction from those with it, will give this knowledge more of the attention it warrants.

The second type of study should investigate what institutional incentives and barriers undergraduate instructors face for implementing cultural competence and sociopolitical consciousness. The theory of practical rationality would call this the institutional obligation, which can enable or constrain teaching activity (Herbst & Chazan, 2012; Shultz, 2020). We suspect there are some systemic changes that need to occur to maximize opportunities for instructors to teach in culturally relevant ways. There is some tension between Ladson-Billings’ (2006) call to customize activities that are relevant to every student and the reality that some of the instructors we interviewed taught large sections, some with over 200 students. Adjunct professors in this study reported high course loads, for example teaching 11 sections interspersed across four institutions. As Morrison et al. (2008) stated, for some instructors the current structure and organization of traditional education can make the task of taking on CRP seem “Herculean” (p. 444). What would happen if departments tasked course coordinators to act as choice architects to make it easier for instructors to access culturally relevant teaching materials? Do instructors have the autonomy to incorporate connections between the content and issues relevant to their students into the curriculum? While we saw some examples of instructors practicing cultural competence among our sample, the manifestations we saw were mostly surface level. For example, showing representation of non-White scientists that are not men may help non-White students that are not men feel an increased sense of belonging, but does not show students how their own backgrounds bring strength to the discipline. A possible study design to examine effective change would look at the implementation of choice architecture in STEM departments. This would involve the creation of resources culturally relevant to instructors’ disciplines and to the student populations at the institution, and collecting data on instructors’ beliefs, their professional obligations, and their subsequent use of the resources provided. By developing the field’s understanding of CR-PCK and levers to encourage enactment of cultural competence and sociopolitical consciousness, future research could continue the work of disrupting existing inequities in STEM education.

## Data Availability

Data sharing is not applicable to this article as no quantitative datasets were generated or analyzed during the current study.

## Appendix

The interview protocol had four sections asking for background information, information about their teaching practice, the culture of their department, and developing their research on teaching. The data for this study came from the second section, which we include below. The text in parentheses was only stated aloud if participants asked for clarification.

### Personal teaching practice

We would like to get a sense of your teaching and thought it might be useful to think about it through the context of an undergraduate course you teach most often or have taught for the longest amount of time. Thinking of that course, what is it? And then address the following questions.

1. What are your goals for students in XXX course? (Academic and non-academic, e.g., communication, collaboration, etc.)
2. What is a specific example of a change you have made to your course (maybe to meet those goal)]?
  - What motivated you to make the change?
  - What impact did the change have on your students? (How do you know…?)
3. How would you change your course, if at all, depending on the majors of students that take your course?
4. Do the identities of students who enroll in that course influence your approach or the way you teach it? If so, how? (e.g., race, gender, parents, business owner, 1st gen student, etc.)
5. How would you describe the “culture” or climate for students in your department in terms of supporting their identities?

- What resources do students have within the department or across the institution that may influence their success?
  1. If you’ve ever taught at another institution that is not an HSI, have you made any changes to how you teach your course since starting at your current institution?
  2. Reflecting on your goals, [insert goals here], how do you assess the impact of your course on students?
  3. If you had more time and sufficient resources, what would you change about your course?
- What is keeping you from making these changes? (e.g., time? Institutional support? Financial resources? Reward structure?)
  2. I want to ask about a few specific reasons for making changes in your course. Here are four:
    1. Needing to address or accommodate the needs of students
    2. Concern for maintaining a good or inclusive classroom environment
    3. Representing the discipline of [chemistry/physics/mathematics/biology] appropriately
    4. Or, finally, requirements for the course from your institution or department

Do any (or all) of these resonate with you? If so, what are some examples of how it informed the way you teach?

## Abbreviations

CRP: Culturally relevant pedagogy
CR-PCK: Culturally relevant pedagogical content knowledge
HSI: Hispanic-Serving Institution
MSI: Minority-Serving Institution
NASEM: National Academies of Sciences, Engineering, and Medicine
STEM: Science, Technology, Engineering, and Mathematics

## Footnotes

1 This theory has been known as the theory of practical rationality *for mathematics teaching (*Chazan et al., 2016; Herbst & Chazan, 2012*)*.

2 In this publication, we capitalize all races, including White, emphasizing that there is no default race and that they are all social constructs with associated sets of cultural practices.

3 Color-evasiveness has more commonly been referred to as color-blindness. Annamma et al. (2015) offered a critique of the term color-blindness in the way the ideology has perpetuated White supremacy. Blindness implies something passively happening to the actor, while evasiveness reflects that the actor is intentionally avoiding addressing race.
